# Tailoring Physicochemical Properties of V_2_O_5_ Nanostructures: Influence of Solvent Type in Sol-Gel Synthesis

**DOI:** 10.3390/ma17102359

**Published:** 2024-05-15

**Authors:** Klaudia Prusik, Daniel Jaworski, Justyna Gumieniak, Agnieszka Kramek, Kamila Sadowska, Marta Prześniak-Welenc

**Affiliations:** 1 Institute of Nanotechnology and Materials Engineering, Gdansk University of Technology, Narutowicza 11/12, 80-233 Gdansk, Poland; s181188@student.pg.edu.pl (K.P.); daniel.jaworski@pg.edu.pl (D.J.); 2 Faculty of Mechanics and Technology, Rzeszów University of Technology, Kwiatkowskiego 4, 37-450 Stalowa Wola, Poland; j.gumieniak@prz.edu.pl (J.G.); a.kramek@prz.edu.pl (A.K.); 3 Polish Academy of Sciences, Ks. Trojdena 4, 02-109 Warsaw, Poland; ksadowska@ibib.waw.pl; 4 Advanced Materials Centre, Gdańsk University of Technology, 80-233 Gdansk, Poland

**Keywords:** vanadium oxide, sol-gel, photocatalysis, adsorption, methylene blue removal

## Abstract

The influence of different solvents, including aqueous and nonaqueous types, on the physicochemical properties of V_2_O_5_ nanostructures was thoroughly investigated. Various characterization techniques, such as XRD, XPS, FTIR, Raman spectroscopy, UV-vis DRS, SEM, TEM, and BET, were employed to analyze the obtained materials. Additionally, the adsorption properties of the synthesized V_2_O_5_ nanostructures for methylene blue were examined, and kinetic parameters of adsorption were calculated. The results demonstrate that the morphology of the obtained crystals can be finely controlled by manipulating water concentration in the solution, showcasing its profound impact on both the structural characteristics and adsorption properties of the nanostructures. Furthermore, the structural changes of the resulting V_2_O_5_ material induced by solvents show strong impacts on its photocatalytic properties, making it a promising photocatalyst.

## 1. Introduction

Vanadium pentoxide, V_2_O_5_, with a band-gap of approximately 2.2 eV shows potential photocatalytic activity in the presence of visible light. However, due to the fast recombination of electron–hole pairs its efficacy in photodegradation reactions is low to moderate, as reported in several papers [1,2,3,4,5,6]. Retardation of the recombination process could significantly elevate V_2_O_5_’s effectiveness in photodegradation reactions. There are three main approaches used to overcome this problem: (*i*) metal doping (Ti, Co, or Zn) into V_2_O_5_ [7,8,9], (*ii*) coupling of two semiconductors, e.g., ZnO-V_2_O_5_, Ce_2_O-V_2_O_5_, etc., [10,11,12,13] or (*iii*) composite manufacturing with reduced graphene oxide (rGO) [6,14,15,16,17]. The summary of studies on the photocatalytic activity of pristine or modified V_2_O_5_ towards methylene blue degradation is presented in Table S1. Analyzing the results presented in the literature (see Table S1), one issue has caught our attention. Different synthesis methods were used to obtain V_2_O_5_ of various size and shape [1,2,3,4,5,6,12,15,18,19,20], but no discussion on their impact on V_2_O_5_’s photocatalytic properties can be found. In new semiconducting materials, defects in crystal lattices are often used as a design parameter for changing the physical properties of the materials [21,22], which is however neglected in studies on V_2_O_5_-based photocatalysts. Defect engineering offers the possible improvement of photocatalyst activity, achieved by enhancing light absorption, optimizing charge transfer and separation, or regulating surface reactions. Recently, Badreldin et al. presented a method for introducing high levels of oxygen vacancies on V_2_O_5_ surfaces. The so called “black V_2_O_5_”, synthesized in the subsequent reduction with NaBH_4_ and thermal treatment (400 °C under argon), showed c.a. 58-fold increase in MB photodegradation efficiency over pristine V_2_O_5_ [23]. However, this is the only report where the influence of intentionally created surface defects on the photocatalytic properties of V_2_O_5_ was mentioned.

Crystal lattice defects disrupt the regular spatial arrangement of atoms within a crystal lattice and, as they naturally occur during the crystal growth process, they can be introduced into V_2_O_5_ crystals by changing synthesis parameters in a controlled manner. In this paper, V_2_O_5_ crystals were obtained in a sol-gel method in two alcohols, namely ethanol or 2-propanol, with or without the addition of water. It is known that the kinetics of the sol-gel reaction strongly depend on the solvent (especially on the presence of water), which in turn has a strong influence on the formation of different defects. Additionally, surface defects can be created in the pre-formed nanocrystals by subsequent thermal treatment, which was also carried out by us. As a result, V_2_O_5_ crystals with surface vanadium species and exposed (001) crystal facets were obtained which showed good adsorption and photocatalytic properties towards methylene blue degradation.

## 2. Materials and Methods

### 2.1. Materials

The vanadium (V) oxytripropoxide (98%, Sigma Aldrich, Saint Louis, MO, USA), anhydrous ethyl alcohol (99.8%, POCH, Gliwice, Poland), anhydrous 2-propanol alcohol (99.5%, Sigma Aldrich, Darmstadt, Germany), acetylacetone (99%, Alfa Aesar, Kandel, Germany), and methylene blue (82%, Chempur, Piekary Slaskie, Poland) were used without further purification. MiliQ water (0.05 µS cm^−1^) was used in all experiments.

### 2.2. Synthesis

The general procedure was repeated after that of [24]. The initial solutions, prepared by combining 0.3 mL of vanadium (V) oxytripropoxide and 0.01 mL of acetylacetone as a catalyst with 1 mL of ethanol (sample E0) or 2-propanol (sample P0), were stirred for 30 min at room temperature (RT). Sample E5 and P5 were obtained in a similar manner, with the addition after 30 min 0.5 mL of water to the ethanolic or 2-propanolic solution of reagents, respectively. Then, all solutions were kept at 50 °C for 48 h in air atmosphere to obtain xerogel powder. Finally, the samples were annealed at 600 °C for 10 h and then cooled to the room temperature.

### 2.3. Structural Characterization

The composition of the acquired samples was analyzed using the X-ray diffraction method (XRD), employing a Philips X’Pert PRO MDP diffractometer system (Royston, UK) with the source based on a Cu anode (λ_Kα1_ = 1.54439 Å). This diffractometer operates utilizing Bragg–Brentano geometry with a θ-θ PW3050/60 goniometer and 1D PIXCEL detector. The measurements were carried out at room temperature, with 2Θ in range of 10–140°, utilizing an acceleration voltage and source current of 40 kV and 30 mA, respectively. The Rietveld refinements of all patterns were performed using HighScore Plus software, version 3.0e. For strain and grains analysis, LaB_6_ was used as a standard. Fourier-transform infrared (FT-IR) spectra were recorded at room temperature using a Perkin–Elmer spectrometer (model Frontier FTIR MIR/FIR, Waltham, MA, USA). The FT-IR spectra of the samples, which were pressed into KBr pellets with a constant material concentration (0.5%), were collected in the wavenumber range of 4000–400 cm^−1^ (mid-IR region) utilizing a KBr beam splitter. The Raman spectra were acquired using a confocal micro-Raman system (Horiba Jobin Yvon, Kioto, Japan) with a 532 nm laser excitation wavelength. Analysis of the samples was conducted using a Thermo Scientific™ K-Alpha™ X-ray Photoelectron Spectrometer (XPS). The X-ray source utilized was an Al Ka micro-focused monochromator with a variable spot size, emitting at an energy level of 1486.68 eV and a power of 360 W. The measurements were performed within a vacuum range of 10^−9^–10^−8^ mbar. The surface morphologies of the samples were examined using a scanning electron microscope (SEM) (FEI Company Quanta FEG 250, Waltham, MA, USA), with the analyzed sample mounted on carbon conductive tape. Transition electron microscopy (TEM) (FEI TECNAI G2 F20, Hillsboro, OR, USA) was used to investigate the crystal morphology and orientation. Nitrogen adsorption–desorption isotherms were assessed using a surface area analyzer NOVAtouch 2, Quantachrome Instruments (Ostfildern, Germany) at 77 K. Prior to the measurements, the samples underwent degassing under dynamic vacuum conditions at 300 °C for 8 h. The specific surface area was determined using the Brunauer–Emmett-Teller (BET) linear equation within the relative pressure range (p/p0) from 0.1 to 0.3. The correlation coefficient for the linear regression was maintained at no less than 0.99. A Zetasizer Nano ZS particle analyzer (manufactured by Malvern Panalytical, Malvern, UK) equipped with a helium–neon laser (with a central wavelength of 632.8 nm and an output power of 4 mW) and a narrowband filter was employed for determining the zeta potentials of the particles through electrophoretic light scattering (ELS). The measurements were conducted at 25 °C using standard, folded capillary cells (DTS1070) in a forward scattering configuration (where scattered light was collected at an angle of 13° with respect to the incident laser beam). Each measurement was carried out five times for accuracy.

The UV–vis reflectance spectra of the designated materials were measured using a UV–vis spectrophotometer Lambda 365+ Perkin-Elmer (Waltham, MA, USA) equipped with a diffuse reflectance accessory. The spectra were recorded over a range of 300–900 nm at a scanning speed of 480 nm min^−1^. Energy band gap values were ascertained by determining the intercept of the tangent from the transformation plot of the Kubelka–Munk function. The calculation of the energy band gap (E_bg_) for the specified powders involved the application of the Kubelka–Munk function (Equation (1)).

(1) $$f(KM)=\frac{{\left(1-R\right)}^{2}}{2R}$$

where *R* is the reflectance.

The band gap was estimated by extrapolation of the linear region of (*f*(*KM*) *hν*)n vs. *hν* to y = 0, where the power “n” depends on the electron transition (n = 1/3, indirect forbidden (i.f.); n = 0.5, indirect allowed (i.a.); n = 2/3, direct forbidden (d.f.); and n = 2, direct allowed (d.a.)) [25].

### 2.4. Adsorption Studies

In the adsorption experiments, 20 milligrams of the acquired samples was combined with 5 mL of an aqueous solution containing methylene blue (MB) with an initial concentration (*C*_0_) of 10^−5^ M in a beaker. The mixture underwent continuous magnetic stirring at 200 rpm, and solutions were probed at consistent time intervals spanning from 0 to 180 min. The alterations in the MB concentration (*C_t_*) during the adsorption process were monitored colorimetrically at a wavelength of 664 nm. The amounts of MB adsorbed onto the samples were calculated by employing Equations (2) and (3).

(2) $$q_{t}=(C_{0}-C_{t})\times \frac{V}{m}$$

(3) $$\%R=\frac{\left(C_{0}-C_{t}\right)}{C_{0}}\times 100$$

where *q_t_* (mg g^−1^) characterizes the adsorption capacity, *C*_0_ (mg L^−1^) is the initial concentration of the adsorbate, *C_t_* (mg L^−1^) corresponds to the adsorbate concentration at time *t* (min), *V* (mL) signifies the volume of the solution, and *m* (mg) denotes the mass of the adsorbent. The removal percentage, *R*, is calculated as a proportion of the initial concentration. The experimental data collected from methylene blue (MB) adsorption experiments were utilized in theoretical modeling to elucidate the kinetics of the process. Nonlinear equations, such as those representing pseudo-first-order (PFO), pseudo-second-order (PSO), Elovich, and the linear equation of intra-particle diffusion (IPD), were chosen to describe and analyze the experimental findings.

### 2.5. Photocatalytic Properties

To evaluate the photocatalytic activity of the powders, the degradation of methylene blue (MB) dye was monitored. In a beaker, 20 milligrams of catalyst was mixed with 50 mL of an aqueous solution of MB (*C*_0_ = 10^−5^ M). Before exposure to light, the suspension underwent magnetic stirring in darkness for 30 min to establish an adsorption/desorption equilibrium. The mixture was exposed to sunlight irradiation using a high-pressure 300 W xenon lamp (LOT–Quantum Design GmbH equipped with an AM1.5G filter) while maintaining constant magnetic stirring at 200 rpm. The intensity of light reaching the solution’s surface was set at 100 mW cm^−2^. Changes in the concentration of MB (*C_t_*) during the decomposition were tracked using a UV–vis spectrophotometer at a wavelength of 664 nm. The kinetics of the photodegradation reaction can be described by the following first order equation:

(4) $$\ln\left(\frac{C_{t}}{C_{0}}\right)=-kt$$

where *C*_0_ (mg/L) is an initial untreated concentration of MB and *C_t_* (mg/L) is a concentration during the photodegradation removal process at a particular time. By plotting ln(*C*/*C*_0_) versus *t*, the reaction rate constant *k* (min^−1^) was determined from the slope of the obtained curves.

To assess the reusability of the synthesized photocatalysts, the cycle experiment for the photodegradation of methylene blue was replicated three times. Following each photodegradation test, the catalyst was collected via centrifugation, washed with pure water under ultrasonication, dried naturally, and subsequently utilized for the next degradation experiment.

## 3. Results

### 3.1. Structural Analysis

#### 3.1.1. XRD Studies and SEM Imaging

**Figure 1:**
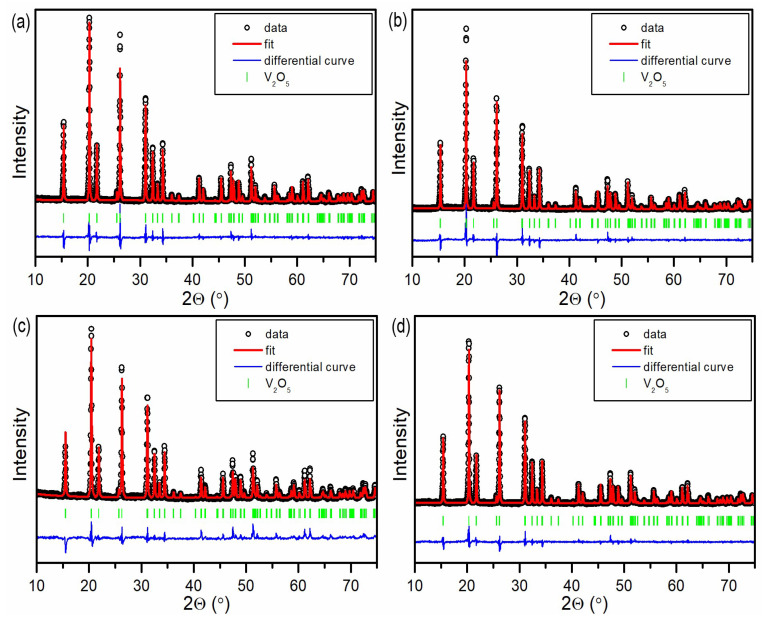
XRD patterns with Rietveld refinement for (**a**) P0, (**b**) P5, (**c**) E0, and (**d**) E5.

The X-ray diffraction patterns were recorded to confirm the phase purity and crystallinity of the obtained samples. Figure 1 illustrates a segment of the XRD patterns (10–80° of 2θ) for the investigated samples. The lattice parameters, volumes, grain sizes, and strains obtained from Rietveld refinement are compiled in Table 1. All prepared samples exhibit a single phase of V_2_O_5_ (COD no. 2020756) with space group #59 (P*mmn*). The differences between lattice parameters in all samples are minimal; however, some changes can be noticed. Values of lattice parameters for E0 are the highest, while the addition of water decreases all lattice parameters except for *c* in E5, which is comparable with E0. Thus, the largest cell belongs to E0 and the smallest to P0. More significant changes are visible in the determined grain sizes and lattice strain. All grains are in the range of several hundred nanometers, and strain is low. The grains in the E0 sample are five times smaller than grains in P0. Additionally, the introduction of water during the synthesis caused an increase in the size of the grains in both cases. In the case of strains, there is no distinct relation. The lattice strain in E0 is smaller than in P0, yet for samples synthesized in an aqueous environment, P5 decreased, while for E5 increased lattice strain was observed. Also worth noting is the significant difference in the intensity of reflexes at 20.3° and 36.1° in samples synthesized with the use of 2-propanol, corresponding to facets (001) and (110) respectively. As observed in the differential curves in Figure 1a,b, in P0, the reflex for the (110) facet is more prominent than expected according to the refinement, whereas in P5, this is true for the reflex for the (001) facet. The ratio between the intensity of reflections of (001) and (110) facets for samples synthesized in 2-propanol is 1.15 and 1.6 for P0 and P5, respectively. Meanwhile, for samples synthesized in ethanol, the ratio is 1.28 and 1.41 for samples E0 and E5, respectively. As can be seen, the addition of water to the synthesis solution affects the relationship between exposed facets and increases the prominence of reflections from the (001) facet. This shows that the formation of specific facets can be tuned by the addition of water into the sol-gel reaction. The manipulation of crystal facets in photocatalysts allows for precise adjustment and optimization of surface atomic arrangements and their corresponding electronic configurations, which affects their photocatalytic performance [21].

**Figure 2:**
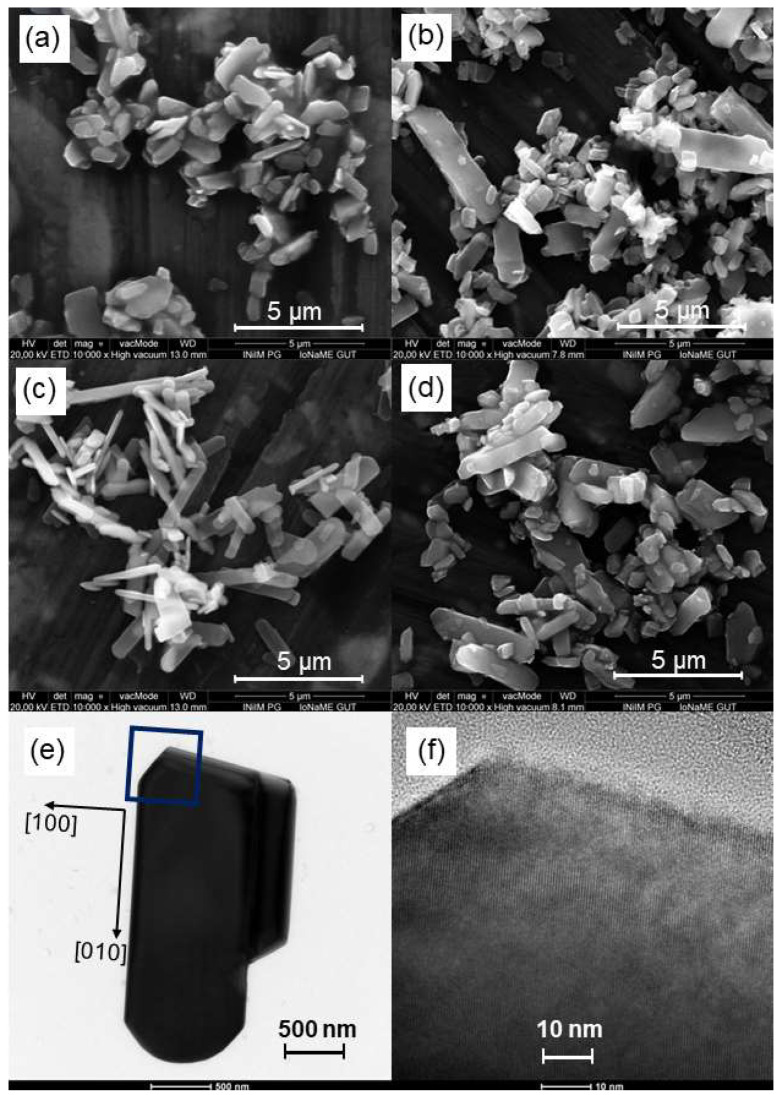
SEM images of (**a**) P0, (**b**) P5, (**c**) E0, and (**d**) E5 samples, and (**e**) TEM and (**f**) high resolution TEM images of sample P0.

The sample morphology was further examined by SEM imaging, and the results are shown in Figure 2a–d. Sample P0 has a belt-like structure with lengths ranging from 1.20 to 5.50 µm, widths between 0.50 and 1.00 µm, and thicknesses ranging from 0.15 to 0.4 µm (Figure 2a). In the case of sample P5 (Figure 2b), noticeable changes in the dimensions compared to those of sample P0 are observed. The addition of water resulted in a decrease in belt length and an increase in width, with the former ranging from 0.20 to 3.00 µm and the latter ranging from 0.15 to 1.20 µm. The crystals of sample E0 (Figure 2c) exhibit a morphology resembling plates with lengths between 0.25 and 2.75 µm, widths between 0.10 and 0.8 µm, and thicknesses between 0.10 and 0.15 µm. When comparing samples E5 (Figure 2d) and E0, more distinct fluctuations in size are observed for the first sample. The crystal length ranges from 0.25 to 5.40 µm, and the width ranges from 0.10 to 1.25 µm, which is particularly noticeable at the upper ends of the ranges.

The atomic arrangement of individual nanocrystals was examined using transmission electron microscopy (TEM) and high-resolution transmission microscopy (HRTEM). In Figure 2e, a TEM image of a typical nanocrystal (sample P0) is shown. Figure 2f displays HRTEM images taken from the area highlighted by the square in Figure 2e. The nanocrystals were observed to grow with their length along the (010) crystallographic direction and their width along the (100) crystallographic direction. The surface of the nanocrystals consists predominantly of (001) atomic planes, and each nanorod is a single crystal.

#### 3.1.2. FTIR and Raman Spectroscopy

**Figure 3:**
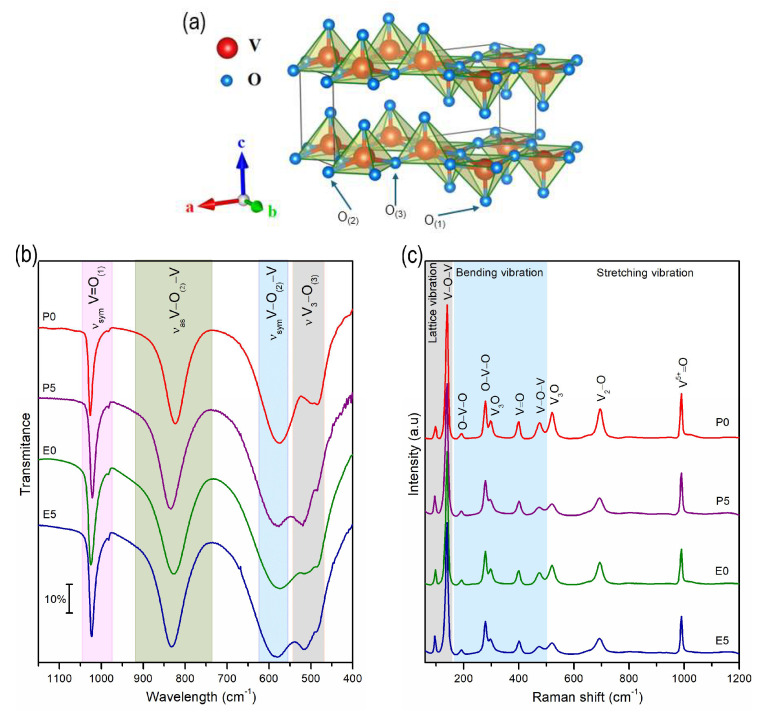
(**a**) Crystal structure of the α-through the Vesta software Ver. 3.5.8 by using the output of Rietveld refinement crystallographic information file [26], and (**b**) FTIR and (**c**) Raman spectra of synthesized samples.

The structure of α-V_2_O_5_ can be described as a succession of V_2_O_5_ chains aligned parallel to the b-axis (Figure 3a). Within each chain segment, two parallel V=O_(1)_ vanadyl bonds are joined by a V–O_(3)_–V bridge, while V–O_(2)_–V bridges serve as links between these segments. Interchain V–O_(2)_ contacts, termed “ladder steps” (LS), establish connections between neighboring V_2_O_5_ chains. Figure 3b,c shows the FTIR and Raman spectra of the analyzed samples, respectively.

Based on the spectroscopic studies outlined in the literature, the absorption band observed in the range of 1025–1018 cm^−1^ (marked as the light pink region in Figure 3b) is associated with the characteristic unshared V=O_(1)_ stretching vibration. Two additional bands, the first falling between 840 and 825 cm^−1^ (green region) and the second between 590 and 570 cm^−1^ (blue region), are attributed to the asymmetric and symmetric stretching vibrations of bridging V–O_(2)_–V, respectively. The band spanning from 530 to 480 cm^−1^ (light grey) corresponds to the edge-sharing V_3_–O_(3)_ stretching modes of the oxygen atoms. The FTIR spectra of the samples synthesized in various solvents exhibit noticeable differences (Figure 3b). To refine the characterization of individual bands, the Gaussian function was employed for curve fitting the FTIR data (refer to ESI Figure S1). The central positions of the bands are shown in Table 2. The symmetric stretching V_3_–O_(3)_ bands for the samples synthesized in a nonaqueous solution (P0 and E0) are shifted to lower frequencies, from the typical band position at 520 cm^−1^ to 485 cm^−1^ [27]. This downshift occurs due to weakening of the V–O–V bond. Furthermore, it is evident in these samples that the relative intensity of the V=O_(1)_ band compared to that of V_3_–O_(3)_ is lower, with values of 0.96 for P0 and 1.02 for E0, compared to those of the samples synthesized in aqueous solutions, P5 and E5, which have values of 1.04 and 1.05, respectively. This decrease in relative intensity, especially for sample P0, could be attributed to interruptions in the V–O–V linkages, resulting in more edge-shearing V–O bonds [28]. In summary, the choice between using aqueous or nonaqueous solutions during the sol-gel synthesis of V_2_O_5_ results in distinct structural perturbations within the matrix.

As can been seen in the Raman spectra (Figure 3c), the significant peak at 140 cm^−1^ is attributed to the skeletal bent vibration, providing evidence for the layered structure of the V_2_O_5_ phase and nanorods [29,30]. Peaks at 191 and 279 cm^−1^ arise from bending vibrations of the O_(3)_–V–O_(2)_ bond related to B2g symmetry [31]. The band at 401 cm^−1^ corresponds to bending vibrations of the V=O bonds associated with Ag symmetry. A signal for the bending vibration of V–O–V doubly coordinated oxygen is observed at 475 cm^−1^. The band at 521 cm^−1^ refers to the stretching modes of triply coordinated oxygen (V_3_–O_(3)_), tied to Ag symmetry vibration, while band at 696 cm^−1^ is associated with the stretching and bending vibrational modes of doubly coordinated oxygen (V_2_–O), related to B2g symmetry [32]. The band at 991 cm^−1^ corresponds to the terminal oxygen (V^5+^–O) stretching mode.

#### 3.1.3. XPS and UV-vis DRS Studies

**Figure 4:**
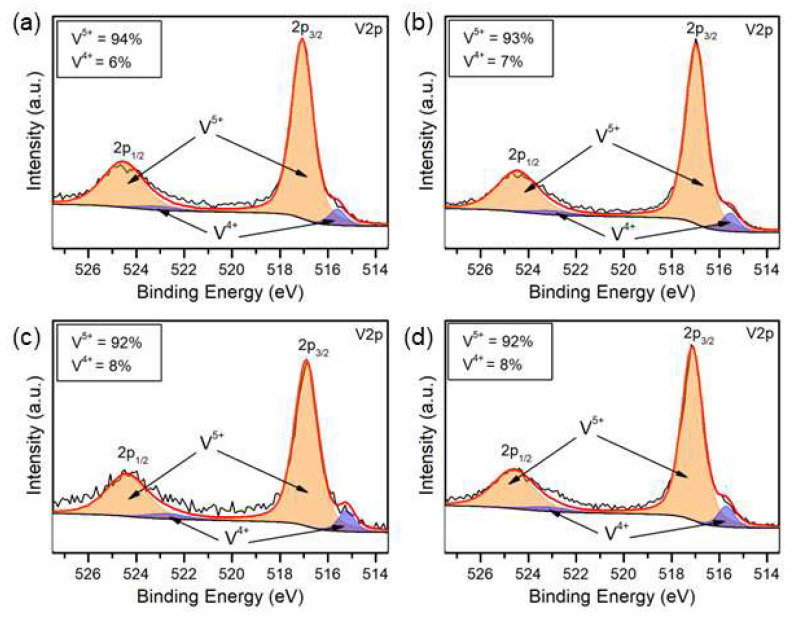
High-resolution V 2p XPS spectra of (**a**) P0, (**b**) P5, (**c**) E0, and (**d**) E5 samples.

It is known that thermal treatment of V_2_O_5_ xerogels introduces surface oxygen vacancies, which was proven by XPS analysis for all four studied samples. The V 2p spectra presented in Figure 4a–d are in agreement with the previous reports for V_2_O_5_ polycrystalline samples [33]. High-resolution XPS spectra of V 2p can be divided into two peaks, located at 517.0 eV and 524.5 eV, referring to V 2p_3/2_ and V 2p_1/2_, respectively. Binding energies of V^4+^ and V^5+^ were positioned at 515.6 eV and 517.5 eV in the V 2p3/2, and at 523 and 524.5 eV in the V 2p^1/2^ [34]. The calculated V^4+^ content was c.a. 6–8%, which is in line with the existing knowledge. Literature findings confirm that, depending on the method of preparation, the amount of V^4+^ can rise up to 10% of the total vanadium ion concentration, especially when the synthesis takes place in an organic solvent [35].

**Figure 5:**
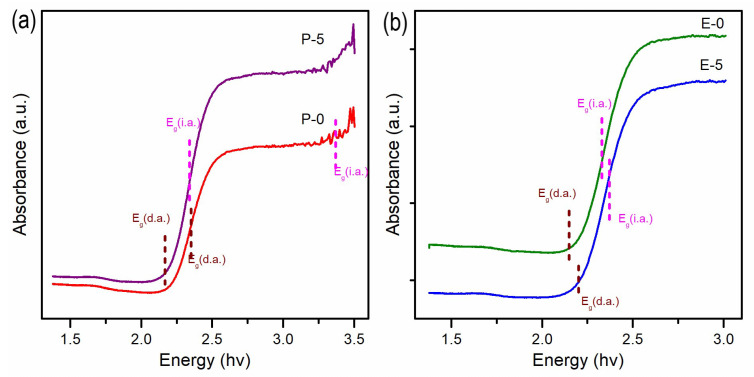
Measured absorbance spectra of (**a**) P0, P5, and (**b**) E0, E-5.

Vanadium pentoxide has a complex electronic structure that is influenced by the crystal phase, quantity of oxygen vacancies, and sample morphology. Bulk α-V_2_O_5_ exhibits a direct bandgap of 2.3–2.4 eV and an indirect bandgap of 1.9–2.0 eV at room temperature. [36]. Changes in optoelectronic properties were reported for V_2_O_5_ of different morphologies. Moreover, the changes in the synthesis conditions deteriorate the stoichiometry of V_2_O_5_’s structure, giving rise to localized states within the gap. For example, the direct band edge of nanoparticles synthesized by a sol-gel method was determined to be 3.27 eV [37]. Li reported [38] a direct band gap of 2.19 eV for a flower-like structure. Puangpetch demonstrated that mesoporous-assembled V_2_O_5_ nanosheets possess a band gap of 2.25 eV [39]. The band gap values of the obtained samples were determined from UV–vis DRS reflection spectra (Figure 5). As observed, the synthesized materials absorb a considerable portion of the light within the visible region. This characteristic proves advantageous for materials utilized in photocatalysis. The reflectance edges observed in both spectra correspond to transitions in the energy band gap, enabling estimation of the energy band gap based on the obtained results. The absorbance spectra of V_2_O_5_ nanorods obtained using different solvents with marked band gaps estimated from the (*f*(*KM*)·*hν*)^n^ vs. *hν* plots are presented in Figure S3. The direct and indirect bandgaps of the samples were calculated and are presented in Table 3. The allowed indirect transitions (i.d.) for all samples are quite similar, ranging between 2.33 and 2.36 eV. On the other hand, the direct energy band gap (d.a.) exhibits slightly more variation among the samples. The lowest energy is observed in sample E0 (2.15 eV), while the highest is in sample P0 (2.23 eV). These discrepancies in the estimated energy bandgaps imply that sample P0 may serve as a superior photocatalyst, owing to its ability to absorb and convert a broader range of electromagnetic radiation.

### 3.2. Adsorption Studies

**Figure 6:**
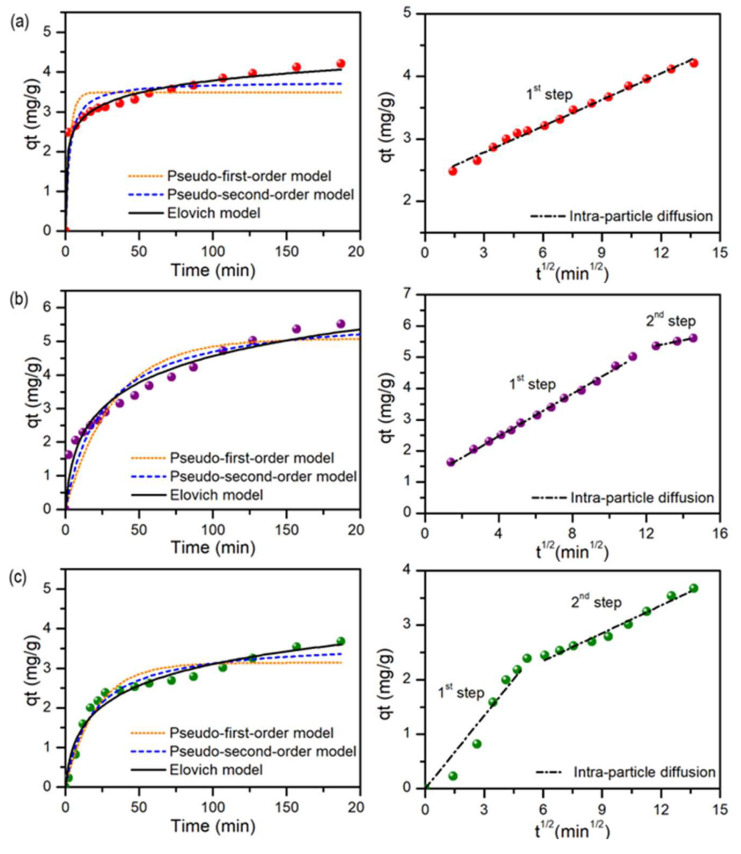

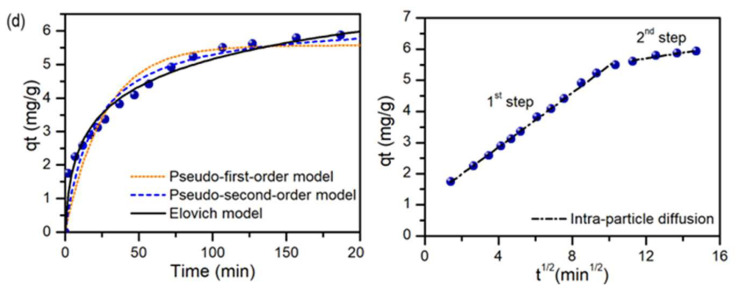
Comparison of adsorption kinetic models fitting for (**a**) P0, (**b**) P5, (**c**) E0, and (**d**) E5.

The first and most important step in the photocatalytic degradation of contaminants is the molecular diffusion and adsorption on the photocatalyst’s surface. Therefore, it is important to elucidate the adsorption kinetics of the studied contaminant onto the studied photocatalysts before photocatalytic experiments. Moreover, thanks to their low solubility, porous structure, and stability against organic dyes, metal oxides are one of the most promising adsorbents for dye removal, as discussed in the recent review [40]. Methylene blue (MB) was chosen as a model compound, as it is frequently used in the literature and thus it is possible to compare the obtained results. Additionally, it is regarded as a contaminant difficult to remove by other methods. In this case, four kinetic models were fitted into the experimental results, as can be seen in Figure 6a–d and in Table 4. Additional info can be found in ESI. For all samples, the best fitting was obtained for the Elovich and intra-particle diffusion models, which both were found to be the most versatile to describe different real adsorption systems, as discussed in some reviews [41,42,43]. According to the recent reports [43,44], the adsorption is dominated by the intraparticle diffusion if the line passes through the origin point (0, 0), which is observed for the E0 sample. In other cases, it is a multiple adsorption process.

**Figure 7:**
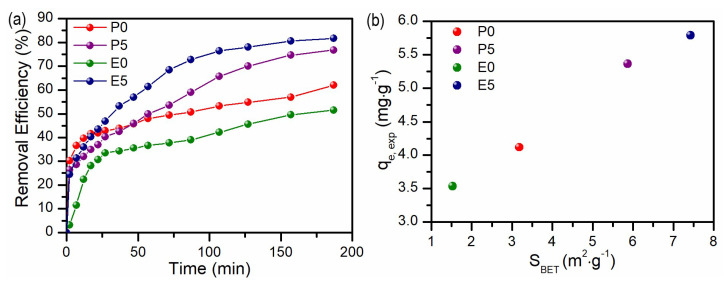
(**a**) Removal efficiency of MB vs. time for studied adsorbents and (**b**) experimental adsorbed quantity (q_e, exp_) vs. surface area (S_BET_) for studied samples.

Figure 7a presents the profiles of MB removal efficiency vs. time for four studied adsorbents. In the discussed plot, two steps can be noticed, especially visible in the case of E0 and P0 samples, where fast adsorption occurred within the first 30 min, followed by a slower, linear increase up to the 180th minute. For samples P5 and E5, similar behavior was observed in the first 30 min, with doubled MB removal in the next 2.5 h as compared to the P0 and E0 samples. Such an observation can be explained by comparing the surface area (S_BET_) of the studied samples (Figure S2). As can be seen in Figure 7 b, the highest MB removal efficiency in the studied period of time was obtained for E5 sample, which has the highest surface area of 7.43 m^2^·g^−1^ and the sample E0 with the lowest S_BET_ = 1.53 m^2^·g^−1^ showed the worst sorption ability under experimental conditions. The measured S_BET_ for samples P0 and P5 were equal to 3.18 and 5.87 m^2^·g^−1^, respectively. For all samples, pore analysis was also performed, revealing the presence of mesopores (see insets in Figure S2). Good linear correlation was obtained between experimental adsorbed quantity (correlated to the removal efficiency) and S_BET_, suggesting that it was the main factor influencing the efficiency of the adsorption process. Additionally, as the MB is a cationic dye, its adsorption was enhanced by the negative charge of the adsorbents. The zeta potential determined for P0 and E0 was equal to −38.8 and −40.9 mV, respectively, and was slightly more negative for the P5 and E5 samples, reaching −46.9 and −49.8 mV, respectively (Figure S4). For all measured samples, a high absolute value of zeta potential assures good dispersibility of the samples in the aqueous medium, which is required for efficient dye removal, either by adsorption or photocatalysis.

### 3.3. Photocatalytic Studies

**Figure 8:**
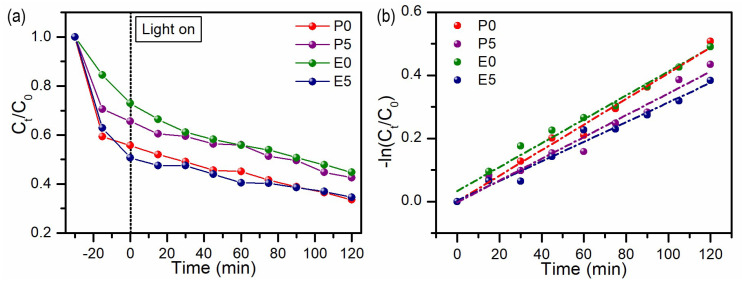
Plot of (**a**) MB photodegradation in time and (**b**) photodegradation kinetics (first-order reaction).

Metal oxides are one of the most promising materials for efficient dye removal from water. Their variety of types and structures makes them a tunable material for photocatalysis with high efficiency towards different dyes’ degradation [45]. Therefore, the photocatalytic properties of the obtained samples were studied, keeping the same experimental conditions as those used for the adsorption experiments. The photodegradation rate was monitored as the change in the MB concentration upon irradiation for 120 min and was measured after 30 min of adsorption/desorption stabilization in the dark. The results are plotted in Figure 8a. Photodegradation can be described by a first-order reaction equation, and the linear relationship between ln(*C_t_*/*C*_0_) and irradiation time is depicted in Figure 7b. The calculated values of the reaction rate constants *k* and regression validation factors (R^2^ and χ^2^_red_) are summarized in Table 5. The assumed degradation mechanism, discussed elsewhere [3], is as follows. V_2_O_5_ particles absorb visible light, producing free electrons in the conduction band and holes in the valence band:

V_2_O_5_ + photon (*hν*) → V_2_O_5_e^−^ (conduction band) + V_2_O_5_h^+^ (valance band)

Oxygen molecules, O_2_, present in the solution react with free electrons in the conduction band to produce superoxide radicals (**∙**O_2_^−^):

V_2_O_5_e^−^(conduction band) + O_2_ → (**∙**O_2_^−^)

Superoxide radicals form H_2_O_2_ by interacting with H^+^ ions. These H_2_O_2_ decompose into (**∙**OH) radicals:

(**∙**O_2_^−^) + H_2_O → H_2_O_2_ + **∙**OH

H_2_O_2_ → 2 **∙**OH

The photogenerated holes react with water molecules to produce (**∙**OH) radicals, which further react with MB, leading to its degradation:

V_2_O_5_h^+^(valance band) + H_2_O → **∙**OH + H^+^

OH + MB → intermediate degradation products (finally CO_2_ + H_2_O+ NH_4_^+^ + NO_3_^−^ + SO_4_^2−^).

An analysis of the results presented in Figure 8 and Table 5 revealed that the highest photocatalytic efficiency was obtained for samples P0 and E5, with MB degradation rates of 67% and 66%, respectively. Comparing the photocatalytic efficiency of a material with literature results is quite complex due to the diversity of studies on dye photodegradation. Table S1 provides a literature summary of studies investigating the photocatalytic properties of photocatalysts based on V_2_O_5_. According to these findings, our results can be compared to those of Le [6], who obtained efficiencies of 58% (180 min) for V_2_O_5_ nanohollows with a surface area of approximately 157 m^2^ g^−1^ and 52% (180 min) for V_2_O_5_ nanospheres with a surface area of 18.6 m^2^ g^−1^. The authors concluded that the high efficiency of photocatalytic activity observed in pure V_2_O_5_ nanostructures is attributed to their high surface area and the high concentration of V^4+^ species on the surface, which is approximately 20%. According to these results, our results can be compared with this experiment. Samples P0 and E5 exhibit significantly lower surface areas of 3.18 m^2^ g^−1^ and 7.43 m^2^ g^−1^, respectively. The concentrations of V^4+^ species in these samples are approximately 6% and 7%, respectively. It can be concluded that the (001) facet also plays a crucial role in the photocatalytic activity of the V_2_O_5_ nanostructures. The relative intensity of this facet is greater for sample P0 than for the other samples, whereas for sample E5, although the relative intensity of the (001) plane is lower, the surface area is more than two times greater.

**Figure 9:**
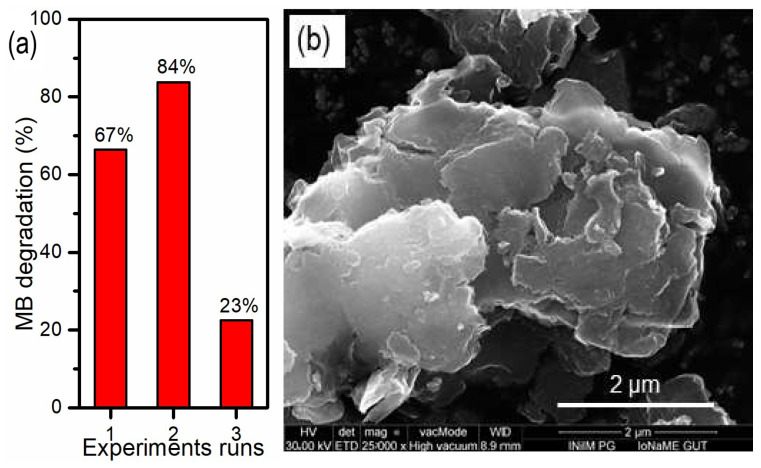
(**a**) Reusability of the P0 sample and (**b**) SEM image of P0 sample after reusability tests.

The reusability of sample P0 was further investigated by conducting three consecutive photodegradation cycles for MB (Figure 9a). The results revealed that the activity of P0 samples increased in the second cycle, but gradually decreased in the third, with the efficiency dropping to 23%. SEM images after the third cycle revealed amorphization of the samples, which may explain the initially better photocatalytic efficiency. This amorphization is associated with an increased surface area and potential damage to the crystal structure, which can positively affect electron–hole separation and inhibit adverse recombination processes. However, subsequent irradiation resulted in more crystal damage and amorphization, leading to lower photocatalytic efficiency. We have shown in our recent paper [46] that mixing reduced graphene oxide with a vanadium-based photocatalyst strongly improves stability towards photodegradation. We also plan to study this behavior in the future for the best photocatalyst obtained in this research.

## 4. Conclusions

The influence of the solvent in the sol-gel synthesis of V_2_O_5_ was investigated to understand its impact on morphology, crystal structure, chemical composition, optical band gap, and adsorption and photocatalytic properties. The results demonstrated that the use of different alcohols (such as ethanol and 2-propanol) and the addition of water to the solution can control the nucleation and growth rate of V_2_O_5_ crystals, thereby determining the possibility of the formation of specific facets, sizes, and shapes. The highest orientation of a particular facet was attained during synthesis in a nonaqueous 2-propanol solution. The addition of water increased the surface area but decreased the crystal orientation of the (001) facet. The highest surface area can be achieved in an aqueous solution, but ethanol is a more suitable alcohol for this purpose. Based on the obtained results, it can be concluded that not only morphology, surface area, and oxygen vacancies but also the distinct (001) facet of the V_2_O_5_ nanostructures are pivotal for photocatalytic activity. Therefore, to enhance the photocatalytic activity of V_2_O_5_, focus should also be directed towards facet engineering.

## Figures and Tables

**Table 1:** The lattice and microstructure parameters derived from Rietveld refinement.

Parameter	P0	P5	E0	E5
a (Å)	3.56447 ± 4.5 × 10^−5^	3.56441 ± 4.4 × 10^−5^	3.56632 ± 8.6 × 10^−5^	3.56524 ± 3.4 × 10^−5^
b (Å)	11.51372 ± 1.51 × 10^−4^	11.51253 ± 1.5 × 10^−4^	11.51837 ± 2.87 × 10^−4^	11.51515 ± 1.15 × 10^−4^
c (Å)	4.37186 ± 6.6 × 10^−5^	4.37147 ± 6.5 × 10^−5^	4.37234 ± 1.23 × 10^−4^	4.37246 ± 5 × 10^−5^
Lattice Strain (%)	0.033	0.023	0.009	0.031
Grains (Å)	4109.1	5993.2	811.5	1719
Volume (Å^3^)	179.4229	179.3847	179.6077	179.5082
**Agreement Indices**				
GOF	4.77	4.15	2.20	1.74
R expected (R_exp_)	6.33	8.52	9.64	9.32
R profile (R_p_)	13.99	17.43	13.69	14.63
Weighted R profile (R_wp_)	13.82	17.36	14.29	12.29

**Table 2:** FTIR band positions and their corresponding assignments.

Vibrational Absorption Band				
Sample	ν_sym_V=O_(1)_	ν_as_V–O_(2)_–V	ν_sym_V–O_(2)_–V	*δ*V_3_–O_(3)_
P0	1027	824	576	485
P5	1021	833	579	520
E0	1024	825	574	485
E5	1022	830	581	517

**Table 3:** Band-edge absorption of the obtained samples.

Eg (eV)	P0	P5	E0	E5
Eg (d.a)	2.23	2.17	2.15	2.20
Eg (i.a)	2.36	2.34	2.33	2.35

**Table 4:** Parameters describing different adsorption kinetic models for analyzed samples.

Parameter	P0	P5	E0	E5
*q_e,exp_*	4.119 ± 0.120	5.363 ± 0.279	3.534 ± 0.102	5.790 ± 0.205
*Pseudo-first-order*				
q_e,est_	3.489 ± 0.123	5.078 ± 0.281	3.142 ± 0.114	5.568 ± 0.224
k_1_	0.322 ± 0.105	0.030 ± 0.005	0.048 ± 0.006	0.038 ± 0.005
R^2^	0.806	0.847	0.938	0.903
χ^2^_red_	0.193	0.362	0.074	0.279
*Pseudo-second-order*				
q_e,est_	3.759 ± 0.114	5.857 ± 0.347	3.688 ± 0.137	6.341 ± 0.258
k_2_	0.100 ± 0.028	0.007 ± 0.002	0.015 ± 0.003	0.008 ± 0.002
R^2^	0.907	0.908	0.968	0.947
χ^2^_red_	0.092	0.219	0.038	0.153
*Elovich*				
α	22.928 ± 6.861	0.569 ± 0.126	0.376 ± 0.080	0.855 ± 0.150
β	2.259 ± 0.101	0.857 ± 0.072	1.246 ± 0.106	0.829 ± 0.049
R^2^	0.991	0.960	0.968	0.978
χ^2^_red_	0.008	0.094	0.037	0.062
*Intraparticle diffusion*				
1st step				
k_i,1_	0.141 ± 0.004	0.342 ± 0.005	0.446 ± 0.025	0.436 ± 0.007
C	2.363 ± 0.029	1.102 ± 0.033	0	1.110 ± 0.043
R^2^	0.991	0.998	0.977	0.997
χ^2^_red_	0.033	0.027	0.337	0.037
2nd step				
k_i,2_	-	0.1203 ± 0.0062	0.169 ± 0.011	0.1203 ± 0.006
C	-	3.8589 ± 0.0850	1.328 ± 0.104	3.859 ± 0.085
R^2^	-	0.995	0.970	0.995
χ^2^_red_	-	0.0001	0.042	0.0001

**Table 5:** Parameters of photocatalytic degradation of MB.

Parameter	P0	P5	E0	E5
k (min^−1^)	0.0041 ± 0.0001	0.0035 ± 0.0002	0.0038 ± 0.0002	0.00312 ± 0.0002
R^2^	0.9893	0.9642	0.9835	0.9741
χ^2^_red_	0.0021	0.0052	0.0028	0.0030
MB degradation (%)	67	58	56	66

## Data Availability

Dataset available on request from the authors.

## Supplementary Materials

The following supporting information can be downloaded at: https://www.mdpi.com/article/10.3390/ma17102359/s1, Adsorption models descriptions (PFO, PSO, Elovich, ID), Figures S1–S4, Table S1.
