# Supplementary material for: Tailoring Physicochemical Properties of V2O5 Nanostructures: Influence of Solvent Type in Sol-Gel Synthesis
Source: Materials (Basel). 2024 May 15;17(10):2359. doi: 10.3390/ma17102359 (PMC11123278; doi:10.3390/ma17102359)
Supplement: Supplementary file 1 [file materials-17-02359-s001.zip › materials-2985938-supplementary.pdf]

Supplementary

Tailoring physicochemical properties of V_2O_5 nanostructures: influence of solvent type in sol-gel synthesis

K. Prusik[a], D. Jaworski[a], J. Gumieniak[c], A. Kramek[c], K. Sadowska[d], M. Prześniak-Welenc[a,b]*

[a]Institute of Nanotechnology and Materials Engineering, Gdansk University of Technology, Narutowicza 11/12, 80-233 Gdansk, Poland
[b] Advanced Materials Centre, Gdańsk University of Technology, 80-233 Gdansk, Poland
[c] Faculty of Mechanics and Technology, Rzeszów University of Technology, Kwiatkowskiego 4, 37-450 Stalowa Wola, Poland
[d]Nalecz Institute of Biocybernetics and Biomedical Engineering, Polish Academy of Sciences, Ks. Trojdena 4, 02-109 Warsaw, Poland

Corresponding author e-mail: marwelen@pg.edu.pl

Pseudo-first-order kinetic model

The pseudo-first-order (PFO), also known as the Lagergren model, is employed to characterize the adsorption mechanism associated with physisorption and diffusion. This model posits the presence of one adsorption site for each adsorbate molecule, and the rate of adsorption is directly proportional to the instantaneous capacity. The nonlinear equations corresponding to this model are provided in eq (ESI-1):

$$q_t = (q_e - e^{-k_1 t}) \qquad \text{ESI-1}$$

where q_t is the adsorption capacity of MB on a adsorbent at a particular contact time, q_e is the adsorption quantity of MB at an equilibrium time, k_1 (min $^{-1}$) is a PFO coefficient and depends upon the initial concentration of adsorbate MB molecules.

Pseudo-second-order kinetic model

The pseudo-second-order (PSO) model postulates the sorption of one molecule onto two active sites of the adsorbent, with the adsorption process being controlled by the chemisorption

mechanism. This mechanism involves valency forces arising from electron sharing or transfer between the adsorbent and the adsorbate. Additionally, the rate of the reaction often relies on the quantity of adsorbate that has been adsorbed. The nonlinear equations associated with this model are presented in eq (ESI-2):

$$q_t = \frac{k_2 q_e^2 t}{1 + k_2 q_e t} \quad \text{ESI-2}$$

here q_t is the adsorption capacity of MB on a adsorbent at a particular contact time, q_e is the adsorption quantity of MB at an equilibrium time, k_2 (mg $g^{-1}min^{-1}$) is a PSO coefficient.

Elovich model

$q_t = (1/\beta) \ln(1 + \alpha\beta t)$

where α and β are constants during any an experiment. The constant α is regarded as the initial rate because (dq_t/dt) approaches α when q_t approaches 0.

Intra-particle diffusion (ID) model has the form:

$q_t = k_p t^{1/2} + C$

k_p is ID rate constant defined in mg/(g·$min^{1/2}$ and C is constant in mg/g.

The magnitude of correlation coefficient (R^2), and Chi-square values (χ^2_{red}) were used for goodness fitting assessment; when R^2 approaches the unity, and the χ^2_{red} aims for the smallest value, the predicted values become closer to the experimental ones. Hence, the corresponding model is most applicable for describing the adsorption system.

All models followed by ((Largitte & Pasquier, 2016; Wang & Guo, 2020, 2022).

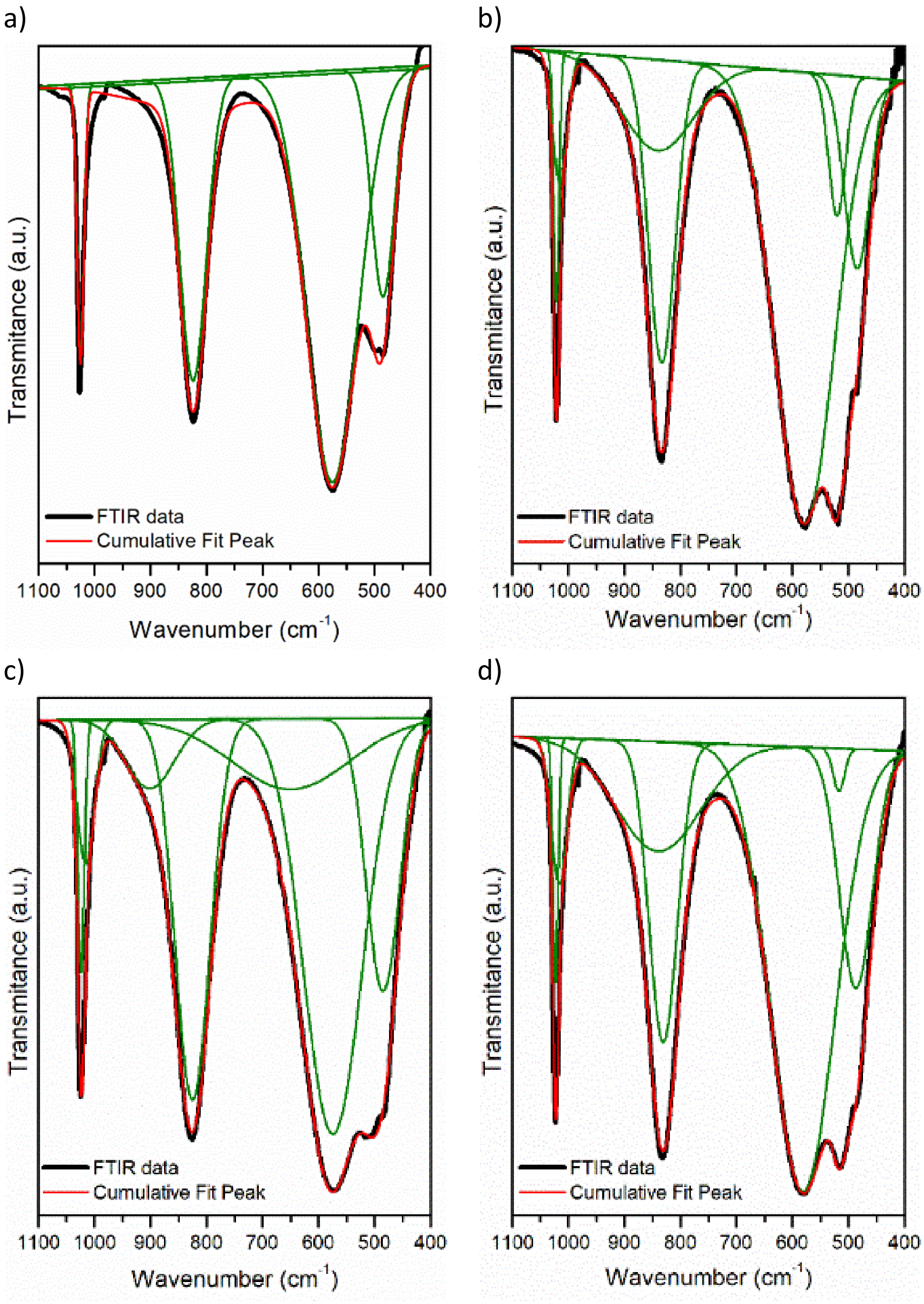

Figure S1. Deconvolution graphics of FTIR spectra with Gaussian bands for sample a) P0, b) P5, c) E0, d) E5

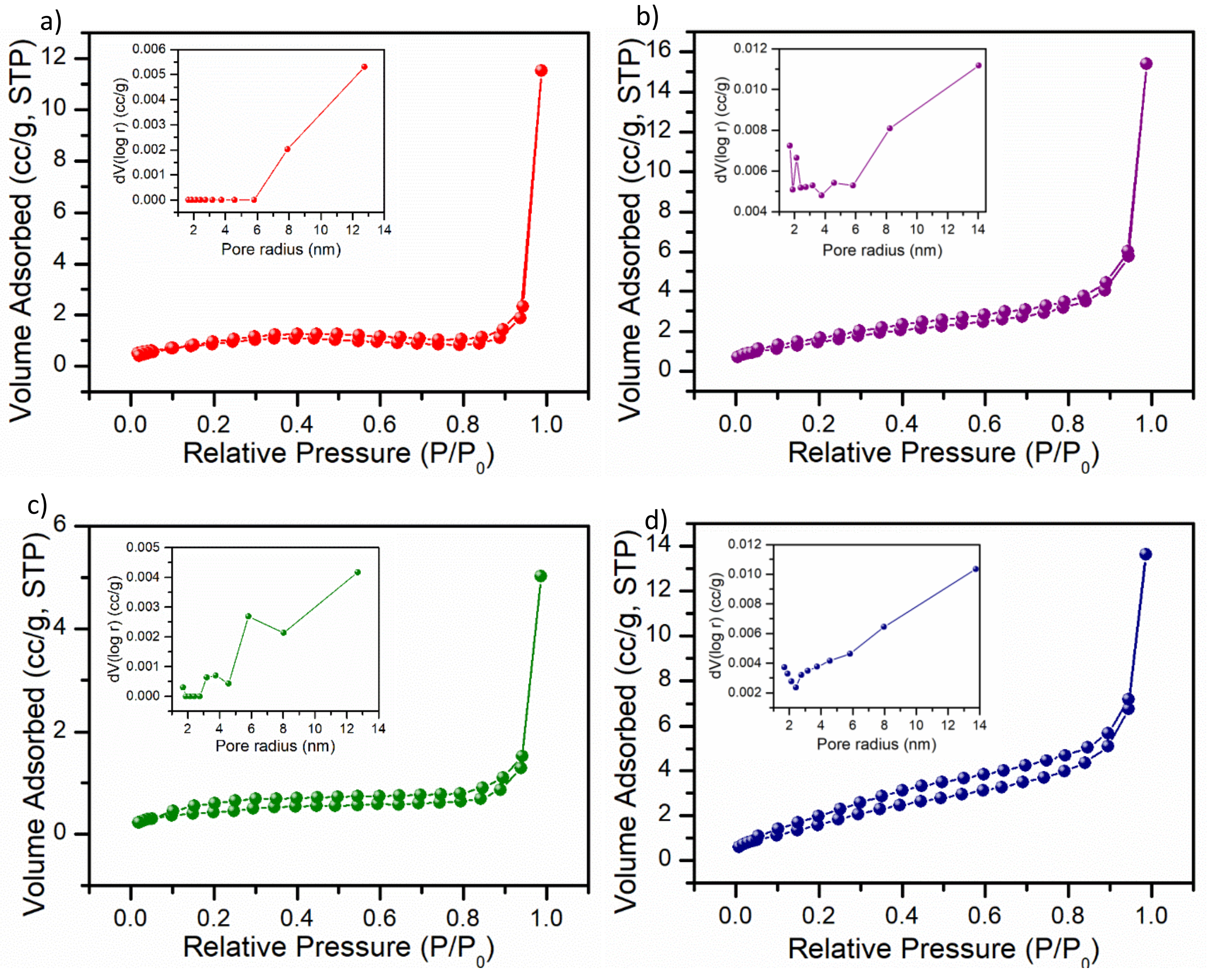

Figure S2. Isotherm and pore size distribution of a) P0, b) P5, c) E0, d) E5 sample

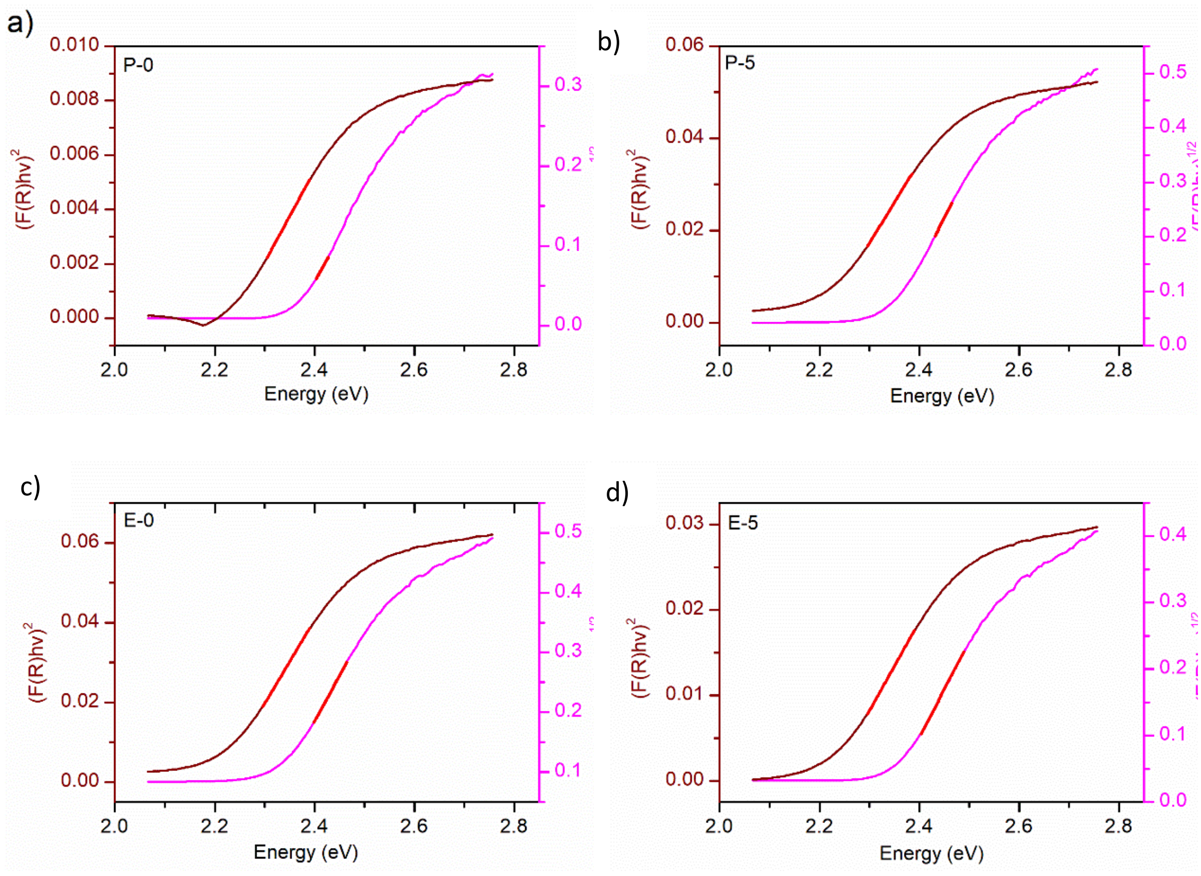

Figure S3. The $(F(R)h\nu)^n$ vs. hν plots (n = 2; 1/2) resulting from the UV-Vis reflectance spectra of a) P0; b) P5; c) E0 and d) E5.

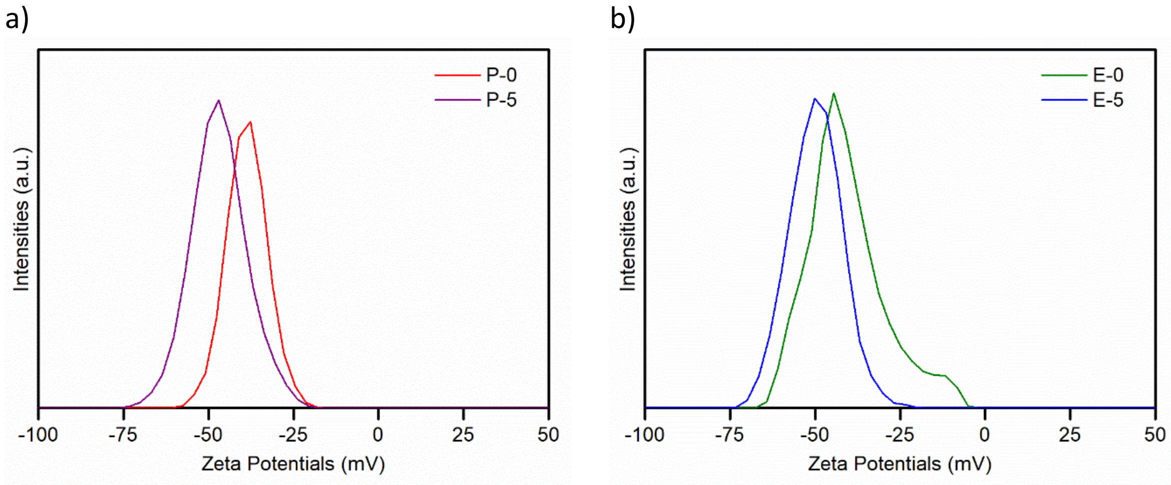

Figure S4. Zeta potential spectra of samples a) P0, P5, and b) E0, E5.

Table S1. Comparison of literature results, where V_2O_5 was used as adsorbent or photocatalyst.

adsorbent type (concentration)	size (nm), shape, (surface area in m^2/g)	C_{dye} (M)	adsorbent conc. and V (mL)	Method	efficiency (%); time (min)	Ref.
alkylimidazolium/V_2O_5 intercalation compounds	-	$3.125 \cdot 10^{-5}$	0.02 mg/mL	adsorption	99 (30)	(Kong et al., 2011)
1D V_2O_5<br><br>V_2O_5+H_2O_2, hydrothermal 200°C, 24 h	(35)	$6.250 \cdot 10^{-5}$	0.15 mg/mL	adsorption<br><br><br>Philips visible light lamps, 6x15 W	17 (210)<br><br><br>31 (210)	(Avansi et al., 2015)
SnO_2@V_2O_5<br><br>TiO_2@V_2O_5<br><br>V chloroalkoxide in oleic acid, heating to 500°C	(70)<br><br>(127)	$1.5 \cdot 10^{-3}$	0.5 mg/mL (4)<br><br>0.5 mg/mL (4)	adsorption	80 (15 min)	(Di Mauro et al., 2019)
V_2O_5<br><br>CeO_2/V_2O_5<br><br>3 h grounded Ce(Ac)2 +NH_4VO_3 annealed at 400°C, 30 min	-	$3 \cdot 10^{-5}$	5 mg/mL<br>(500)	visible light irradiation (projection lamp 7748XHP 250 W, Philips, 532 nm)	27.5 (210)<br><br>64.2 (210)	(Saravanan et al., 2013)
ZnO/V_2O_5<br><br><br>NH_4VO_3+$Zn(Ac)_2$ calcined at 450 °C for 30 min		500 mg in unknown volume	500 mg of catalyst in 500 mg of MB aqueous solution	visible light exposure	97 (120)	(Saravanan et al., 2014)

10%Co-V_2O_5 nanoparticles<br><br>NH_4VO_3+ CoSO4·7H_2O oxalic acid+SDS	-	10^{-4}	0.5 mg/mL	visible source (λ > 420 nm)	99 (105)	(Suresh et al., 2014)
flake-type structure of V_2O_5-ZnO (V_2O_5+HCl)	-	not given	not given	visible light exposure	97 (80)	(Shukla & Shukla, 2018)
V_2O_5 nanorods<br><br>NH_4VO_3 + H_2SO_4	(11)	$5 \cdot 10^{-5}$	0.2 mg/mL (100)	visible light exposure	24 (300)	(Jayaraj et al., 2018)
V_2O_5 nanohollows<br><br>V_2O_5 nanospheres<br><br>rGO-V_2O_5 nanohollows<br><br>rGO- V_2O_5 nanospheres<br><br>NH_4VO_3+hydrazine	(157.4)<br><br>(18.6)<br><br>(206.3)<br><br>(24.5)	$1.5 \cdot 10^{-5}$	5 mg on quartz substrate immersed in 30 mL	Xe-lamp irradiation	58 (180)<br><br>52 (180)<br><br>90 (180)<br><br>80 (180)	(Le et al., 2019)
rGO-V_2O_5 nanorods<br><br>NH_4VO_3+H_2SO_4, hydrothermal 150°C, 18 h calcined at 400°C for 90 min		$1.6 \cdot 10^{-4}$	0.1 mg/mL (100)	three 40 W visible lamps, 450–700 nm<br><br>three 8 W UV lamps, 360 nm<br><br>50 W mercury-lamp, 400 nm	60 (390)<br><br>70 (210)<br><br>85 (255)	(Aawani et al., 2019)
rGO-V_2O_5 composite	-	$3.125 \cdot 10^{-5}$	0.3 mg/mL (20)	40 W tube light	71 (20)	(Mishra et al., 2020)

V_2O_5<br>NH_4VO_3 +citric acid					31 (20)	
V_2O_5_PVA<br><br>V_2O_5_SDS<br><br>V_2O_5_T100<br><br>V_2O_5_T80<br><br>NH_4VO_3 +H_2O+EtOH + surfactant sodium dodecyl sulphate (SDS), Tween 80, Triton X-100 (T100) and polyvinyl alcohol (PVA)	(1.18)<br><br>(3.13)<br><br>(1.73)<br><br>(2.10)	$3.125 \cdot 10^{-5}$	1 mg/mL (10)	four UV-A Sylvania TLD 15W/08 lamps (Wilmington, MA, USA) (350–390 nm, 0.5 mW/cm^2)	5 (180)<br><br>42 (180)<br><br>0 (180)<br><br>60 (180)	(Ibrahim et al., 2020)
black V_2O_5<br><br>V_2O_5<br><br>NH_4VO_3 calcination + reduction with $NaBH_4$	(3.0)<br><br>(3.2)	0.0625	0.5 mg/mL (50)	artificial solar simulator 700 W/m^2<br><br>75.6 mg/L adsorption of MB after 3 h in dark	93 (60)<br><br>10 (60)	(Badreldin et al., 2021)
V_2O_5 nanoparticles<br><br>$NaVO_3$+NH_4Cl+CTAB	-	not given	adequate amount (100 mL)	Xe arc lamp (300 W)<br><br>5% adsorption after 30 min in dark	92 (90)	(Jenifer et al., 2021)
V_2O_5<br><br>g-C_3N_4-Co_3O_4-V_2O_5<br><br>$VOCl_3$ + NH_3	-	$1.6 \cdot 10^{-4}$	fixed amount (100)	halogen light source 500 W	48 (90)<br><br>93 (90)	(Palanisamy et al., 2021)
V_2O_5	-	not given	0.25 mg/mL	visible light exposure	76 (70)	(Babar et al., 2020)

NH_4VO_3+NH_3<br><br>dried 400°C for 2 h						
V_2O_5<br><br>rGO-V_2O_5 composite<br><br>NH_4VO_3 + HCl+ polyethylene glycol	(2.72)<br><br>(3.97)	10^{-5}	0.4 mg/mL (500)	visible light exposure	60 (105)<br><br>90 (105)	(Sharma et al., 2022)
V_2O_5<br><br>5%Ti-V_2O_5<br><br>NH_4VO_3+Ti(OC_3H_7)$_4$ in polyethylene glycol, 24 h, 165°C		10^{-5}	0.2 mg/mL (10)	visible light irradiation	65 (90)<br><br>82 (90)	(Neelima et al., 2022)
V_2O_5 layers on glass<br><br>V_2O_5+H_2O_2 24 h of aging,<br><br>calcined at 500°C, 30 min		$1.6\cdot10^{-5}$	2.5x7.5 cm glass covered with two layers of V_2O_5	35-W Xe lamp for 12 h	85 (720)	(Alfaro Cruz et al., 2022)
Zn-doped V_2O_5<br><br>V_2O_5<br>$NaVO_3$ + NH_4Cl+CTAB+ Zn(Ac)$_2$<br>600°C for 4 h	(2.62)<br><br>(2.27)	not given	not given (60)	Xe arc lamp 300 W	99 (90 min)<br><br>94 (105 min)	(Jenifer & Sriram, 2023)
1:1 rGO-V_2O_5 composite<br><br>V_2O_5<br>NH_4VO_3+oxalic acid	-	$3.125\cdot10^{-5}$	0.3 mg/mL (20)	room lighting	94 (8)<br><br>15 (22)	(Neha et al., 2024)
V_2O_5 (sample P0)	(3.18)	10^{-5} M	0.4 mg/ml	Xe arc lamp 300 W, 100 mW cm^{-2}	67 (180)	This work

V_2O_5 (sample P5)	(5.87)	10^{-5} M	0.4 mg/ml	Xe arc lamp 300 W, 100 mW cm^{-2}	58 (180)	This work
V_2O_5 (sample E0)	(1.53)	10^{-5} M	0.4 mg/ml	Xe arc lamp 300 W, 100 mW cm^{-2}	56 (180)	This work
V_2O_5 (sample E5)	(7.43)	10^{-5} M	0.4 mg/ml	Xe arc lamp 300 W, 100 mW cm^{-2}	66 (180)	This work

Aawani, E., Memarian, N., & Dizaji, H. R. (2019). Synthesis and characterization of reduced graphene oxide–V2O5 nanocomposite for enhanced photocatalytic activity under different types of irradiation. *Journal of Physics and Chemistry of Solids*, *125*, 8–15. https://doi.org/10.1016/j.jpcs.2018.09.028

Alfaro Cruz, M. R., Vázquez G., L. F., Garay-Rodríguez, L. F., & Torres-Martínez, L. M. (2022). Hierarchical V2O5 thin films and its photocatalytic performance. *Materials Letters*, *324*. https://doi.org/10.1016/j.matlet.2022.132751

Avansi, W., De Mendonça, V. R., Lopes, O. F., & Ribeiro, C. (2015). Vanadium pentoxide 1-D nanostructures applied to dye removal from aqueous systems by coupling adsorption and visible-light photodegradation. *RSC Advances*, *5*(16), 12000–12006. https://doi.org/10.1039/c4ra12788a

Babar, B. M., Mohite, A. A., Patil, V. L., Pawar, U. T., Kadam, L. D., Kadam, P. M., & Patil, P. S. (2020). Sol-gel prepared vanadium oxide for photocatalytic degradation of methylene blue dye. *Materials Today: Proceedings*, *43*, 2673–2677. https://doi.org/10.1016/j.matpr.2020.04.205

Badreldin, A., Imam, M. D., Wubulikasimu, Y., Elsaid, K., Abusrafa, A. E., Balbuena, P. B., & Abdel-Wahab, A. (2021). Surface microenvironment engineering of black V2O5 nanostructures for visible light photodegradation of methylene blue. *Journal of Alloys and Compounds*, *871*. https://doi.org/10.1016/j.jallcom.2021.159615

Di Mauro, A., Landström, A., Concina, I., Impellizzeri, G., Privitera, V., & Epifani, M. (2019). Surface modification by vanadium pentoxide turns oxide nanocrystals into powerful adsorbents of methylene blue. *Journal of Colloid and Interface Science*, *533*, 369–374. https://doi.org/10.1016/j.jcis.2018.08.070

Ibrahim, I., Belessiotis, G. V., Arfanis, M. K., Athanasekou, C., Philippopoulos, A. I., Mitsopoulou, C. A., Romanos, G. E., & Falaras, P. (2020). Surfactant effects on the synthesis of redox bifunctional v2o5 photocatalysts. *Materials*, *13*(20), 1–13. https://doi.org/10.3390/ma13204665

Jayaraj, S. K., Sadishkumar, V., Arun, T., & Thangadurai, P. (2018). Enhanced photocatalytic activity of V2O5 nanorods for the photodegradation of organic dyes: A detailed understanding of the mechanism and their antibacterial activity. *Materials Science in Semiconductor Processing*, *85*, 122–133. https://doi.org/10.1016/j.mssp.2018.06.006

Jenifer, A., Sastri, M. L. S., & Sriram, S. (2021). Photocatalytic dye degradation of V2O5 Nanoparticles—An experimental and DFT analysis. *Optik*, *243*. https://doi.org/10.1016/j.ijleo.2021.167148

Jenifer, A., & Sriram, S. (2023). Enhanced photocatalytic organic dye degradation activities of pristine and Zn-doped V2O5 nanoparticles. *Applied Surface Science*, *611*. https://doi.org/10.1016/j.apsusc.2022.155629

Kong, A., Ding, Y. J., Wang, P., Zhang, H. Q., Yang, F., & Shan, Y. K. (2011). Novel alkylimidazolium/vanadium pentoxide intercalation compounds with excellent adsorption performance for methylene blue. *Journal of Solid State Chemistry*, *184*(2), 331–336. https://doi.org/10.1016/j.jssc.2010.12.008

Largitte, L., & Pasquier, R. (2016). A review of the kinetics adsorption models and their application to the adsorption of lead by an activated carbon. *Chemical Engineering Research and Design*, *109*, 495–504. https://doi.org/10.1016/j.cherd.2016.02.006

Mishra, A., Panigrahi, A., Mal, P., Penta, S., Padmaja, G., Bera, G., Das, P., Rambabu, P., & Turpu, G. R. (2020). Rapid photodegradation of methylene blue dye by rGO- V2O5 nano composite. *Journal of Alloys and Compounds*, *842*. https://doi.org/10.1016/j.jallcom.2020.155746

Neelima, M., Vandana, S., Kathirvel, A., Sivakumar, M., & Maheswari, A. U. (2022). Titanium doped V2O5 nanostructures by chemical synthesis for photocatalytic performance enhancement. *Optik*, *252*. https://doi.org/10.1016/j.ijleo.2021.168516

Neha, Turpu, G. R., Das, P., Seo, Y. S., Rabani, I., & Reddy, S. S. K. (2024). Ultrafast photodegradation of methylene blue dye and supercapacitor applications of flower like hydrothermal synthesized V2O5 nano -structures on rGO as nano - composite. *Journal of Physics and Chemistry of Solids*, *184*. https://doi.org/10.1016/j.jpcs.2023.111673

Palanisamy, G., Bhuvaneswari, K., Srinivasan, M., Vignesh, S., Elavarasan, N., Venkatesh, G., Pazhanivel, T., & Ramasamy, P. (2021). Two-dimensional g-C3N4 nanosheets supporting Co3O4-V2O5 nanocomposite for remarkable photodegradation of mixed organic dyes based on a dual Z-scheme photocatalytic system. *Diamond and Related Materials*, *118*. https://doi.org/10.1016/j.diamond.2021.108540

Saravanan, R., Gupta, V. K., Mosquera, E., & Gracia, F. (2014). Preparation and characterization of V2O5/ZnO nanocomposite system for photocatalytic application. *Journal of Molecular Liquids*, *198*, 409–412. https://doi.org/10.1016/j.molliq.2014.07.030

Saravanan, R., Joicy, S., Gupta, V. K., Narayanan, V., & Stephen, A. (2013). Visible light induced degradation of methylene blue using CeO 2/V2O5 and CeO2/CuO catalysts. *Materials Science and Engineering C*, *33*(8), 4725–4731. https://doi.org/10.1016/j.msec.2013.07.034

Sharma, D., Faraz, M., Kumar, D., Takhar, D., Birajdar, B., & Khare, N. (2022). Visible light activated V2O5/rGO nanocomposite for enhanced photodegradation of methylene blue dye and photoelectrochemical water splitting. *Inorganic Chemistry Communications*, *142*. https://doi.org/10.1016/j.inoche.2022.109657

Shukla, P., & Shukla, J. K. (2018). Facile sol-gel synthesis and enhanced photocatalytic activity of the V2O5-ZnO nanoflakes. *Journal of Science: Advanced Materials and Devices*, *3*(4), 452–455. https://doi.org/10.1016/j.jsamd.2018.09.005

Suresh, R., Giribabu, K., Manigandan, R., Munusamy, S., Praveen Kumar, S., Muthamizh, S., Stephen, A., & Narayanan, V. (2014). Doping of Co into V2O5 nanoparticles enhances photodegradation of methylene blue. *Journal of Alloys and Compounds*, *598*, 151–160. https://doi.org/10.1016/j.jallcom.2014.02.041

Wang, J., & Guo, X. (2020). Adsorption kinetic models: Physical meanings, applications, and solving methods. In *Journal of Hazardous Materials* (Vol. 390). Elsevier B.V. https://doi.org/10.1016/j.jhazmat.2020.122156

Wang, J., & Guo, X. (2022). Rethinking of the intraparticle diffusion adsorption kinetics model: Interpretation, solving methods and applications. *Chemosphere*, *309*. https://doi.org/10.1016/j.chemosphere.2022.136732
